# Investigation of the Electronic Structure and Optical Spectra of Uranium (IV), (V), and (VI) Complexes Using Multiconfigurational Methods

**DOI:** 10.1021/acs.jpca.2c03314

**Published:** 2022-09-06

**Authors:** Michael Godsall, Nicholas F. Chilton

**Affiliations:** Department of Chemistry, The University of Manchester, Manchester M13 9PL, U.K.

## Abstract

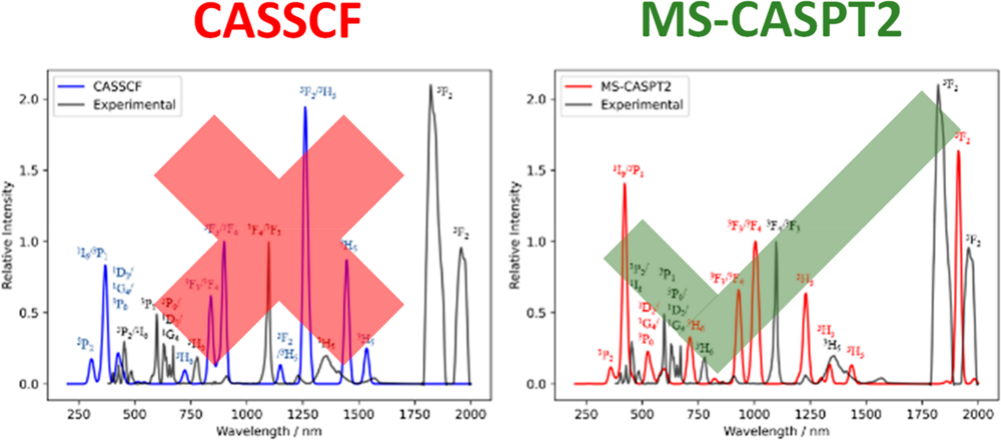

Interpreting electronic spectra of uranium-containing compounds is an important component of fundamental chemistry as well as in the assessment of waste streams in the nuclear fuel cycle. Here we employ multiconfigurational calculations with CASSCF or DMRGSCF methods on exemplar uranium molecules [U^VI^O_2_Cl_4_]^2–^, [U^V^(TREN^TIPS^)(N)]^−^, and [U^IV^Cl_5_(THF)]^−^, featuring an array of geometries and oxidation states, to determine their effectiveness in predicting electronic spectra, compared to literature calculations and experimental data. For [U^VI^O_2_Cl_4_]^2–^, DMRGSCF alone shows poor agreement with experiment, which can be improved by adding corrections for dynamic correlation with MC-PDFT to give results of similar quality to TD-DFT. However, for [U^V^(TREN^TIPS^)(N)]^−^ the addition of dynamical correlation via MC-PDFT or CASPT2 made no improvements over CASSCF, suggesting that perhaps other factors such as solvation effects could be more important in this case. Finally, for [U^IV^Cl_5_(THF)]^−^, dynamical correlation included via MS-CASPT2 on top of CASSCF calculations is crucial to obtaining a quantitatively correct spectrum. Here, MC-PDFT fails to even qualitatively describe the spectrum, highlighting the shortcomings of single-state methods in cases of near-degeneracy.

## 1 Introduction

The decommissioning of legacy nuclear reactor sites in the U.K., such as Sellafield, is an expensive and time-consuming task.^1^ Due to poor record keeping, the composition of the waste stored at such facilities is mostly unknown. Hence, the determination of the chemical species (speciation) in legacy wastes is of crucial importance, and an effective proposed technique is luminescence spectroscopy.^2^ Luminescence spectroscopy is ideal in this case, as the nature of the emission spectra of actinide compounds carries an imprint of both electronic and vibrational states of the molecules within the waste, providing information on elements, oxidation states, and chemical structure. Through comparison to a library of luminescence spectra, the experimental spectrum of a mixture could thus be deconvoluted. While a good idea in theory, the preparation of a range of model compounds in different conditions is complicated due to radiolysis and changes in oxidation state due to disproportionation^3^ or other redox processes. Thus, one route would be to generate an accurate library of luminescence spectra using computational methods.

To accurately calculate a theoretical luminescence spectrum, one must accurately determine both the electronic structure and the vibrational spectrum of the complex (for both ground and excited states) as well as account for an accurate representation of the molecular environment (e.g., solution, surface immobilization, or solid). When it comes to actinide complexes, the confluence of a large number of electrons, strong relativistic effects, orbital degeneracy, and accessible redox states makes even the first step (an accurate calculation of electronic structure) difficult. There are several approaches to the calculation of electronic structure and excitation energies, but by far the most utilized method is time-dependent density functional theory (TD-DFT), because it circumvents the calculation of excited state wave functions and instead uses the change in electronic density in response to an external potential to derive excited-state energies,^4^ so it is therefore relatively fast. Indeed, it can also be reasonably accurate depending on the functional chosen, such as the long-range corrected functional CAM-B3LYP.^5^ However, a poor choice of functional can also lead to poor results, so calculations are usually tailored to specific molecules and therefore one cannot use such methods to generate a reliable spectral reference library. DFT is also inherently a single-configuration approach and therefore cannot account for orbital degeneracy effects which are common in both ground and excited states of actinide complexes.

An alternative strategy is to use explicit wave function-based methods, which also allow a more rigorous inclusion of spin–orbit coupling which plays a crucial role in the electronic spectra of actinide complexes. As we wish to calculate both ground- and excited-state properties as well as account for orbital degeneracy, this necessitates a multireference method. A very common choice is the complete active space self-consistent field (CASSCF) method,^6^ which treats a subset of the molecular orbital space with full configuration interaction (FCI) and the remaining orbitals with Hartree–Fock theory.^7^ Such methods have found extensive use for probing actinide chemistry; examples include Vallet et al. investigating the mechanism of water exchange of actinyl aquo ions,^8^ Gagliardi et al. and Heit et al. calculating electronic structure and spectra,^9−11^ Mounce et al. interpreting nuclear magnetic resonance data,^12,13^ and Autschbach et al. predicting magnetic properties.^14^ Theoretical methods are also able to probe the limits of our understanding of bonding at the bottom of the periodic table, exemplified by the definition of a quintuple bond in U_2_ by Roos et al.^15^ and recently countered to in fact be a quadruple bond when relativistic effects are considered more rigorously.^16^

Using CASSCF allows the inclusion of the “important” orbitals in optical excitation, without the computational burden of FCI in the entire orbital space. The advantage of this method over TD-DFT is the inclusion of static correlation as well as relativistic effects. However, CASSCF suffers from a lack of dynamical correlation (DFT attempts to include some dynamic correlation effects with the exchange-correlation functional) and thus often needs to be corrected using perturbative methods such as complete active space second-order perturbation theory (CASPT2)^17^ or the more recent multiconfiguration pair-density functional theory (MC-PDFT).^18^ The cost of the calculations also increases very quickly when the active space is expanded, though this can be alleviated by using the restricted active space self-consistent field (RASSCF) methods to limit the number of excitations or, alternatively, an approximate CI solver such as a density matrix renormalization group (DMRG)^19−21^ can be used for much larger active spaces. Unlike CASSCF, DMRG uses matrix product states allowing the number of configurations to be reduced by reducing the size of the matrices via a single-value decomposition. However, all of these multiconfigurational methods are far from the “black-box” approach of DFT methods, and results can vary significantly given details of the active space and dynamic correlation corrections.

Thus, we set out to systematically compare TD-DFT, CASSCF, DMRG, CASPT2, and MC-PDFT methods for calculating the excitation spectra of some uranium complexes. We find that TD-DFT and DMRG-MC-PDFT methods seem to be appropriate for uranyl U(VI) compounds, while we find that minimal CASSCF with or without CASPT2 corrections seems most appropriate for U(V) and U(VI) compounds. However, we caution that the representation of the environment of the uranium compound, in this case, the solvent, must be crucial to obtaining experimental accuracy and hence building libraries of spectra.

## 2 Methods

DFT optimizations were performed in the gas phase using *Gaussian 09*(22) with the PBE0 functional^23^ and the D3 version of Grimme’s dispersion.^24^ The Stuttgart RSC 1997^25−28^ basis set and ECP were used for uranium, and the cc-pVTZ basis set^29^ was used for all other atoms. Vibrational frequency calculations were performed with the HPModes option. CASSCF calculations were performed using OpenMolcas 19.11^30,31^ with the geometries obtained from DFT optimization. ANO-RCC-VTZP, VDZP, and VDZ basis sets^32,33^ were used for the uranium, first coordination sphere, and other atoms, respectively. Cholesky decomposition of the two-electron integrals at a threshold of 10^–8^ was used for all calculations. The spin–orbit coupling and dipole transition strengths were calculated using the RASSI module. DMRGSCF calculations were performed using the QCMaquis DMRG program suite^19−21^ with a maximum bond dimension of 512. Dynamical correlation was added using either singlet-state CASPT2 (SS-CASPT2),^17^ multistate CASPT2 (MS-CASPT2),^34^ extended multistate CASPT2 (XMS-CASPT2),^35^ or MC-PDFT.

## 3 Results and Discussion

### 3.1 [UO_2_Cl_4_]^2–^ – U(VI)

**Figure 1:**
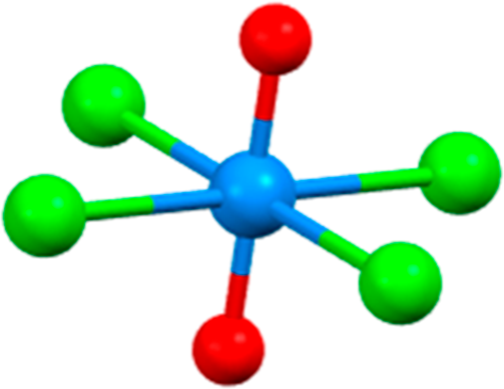
Molecular structure of [UO_2_Cl_4_]^2–^. Red = oxygen, green = chlorine, and blue = uranium.

Uranium is commonly found in the +6 oxidation state as uranyl compounds in environmental settings and as such is an important class of molecules to consider. Uranium(VI) compounds are 5f^0^; therefore, optical transitions arise from ligand–metal and/or metal–ligand charge-transfer excitations. The [UO_2_Cl_4_]^2–^ anion (Figure 1) has been investigated both experimentally and computationally, so it is a good benchmark system for our purposes. In this compound, excitation is supposed to occur from the ground singlet state (S0) to the first excited triplet state (T1) from which emission occurs back to S0 after internal conversion. Pierloot, Van Besien, and colleagues found good agreement between their SOC-CASPT2 excitation energies and experimental data, thus they were able to show that the excitation occurs from a σ_u_ orbital (S0) to a nonbonding 5f_δ_ orbital (T1) and that two-component TD-DFT approaches were also quite accurate.^36,37^ Tecmer et al.^38^ and most recently Oher et al.^39^ have also shown that TD-DFT with the CAM-B3LYP functional is quite accurate compared to SOC-CASPT2, SOC-CI, and experimental data, agreeing on the nature of the excitation. Here we sought to use larger active spaces to better treat electron correlation in the optical excitation and emission processes.

**Figure 2:**
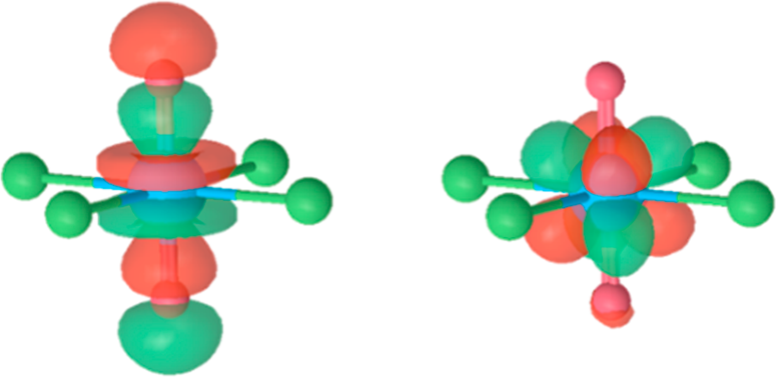
(Left) Highest occupied orbital in the ground state S0 (σ_u_) and (right) the newly singly occupied molecular orbital in the first excited state T1 (5f_δ_) for [UO_2_Cl_4_]^2–^.

We have optimized geometries for the singlet ground state and first excited triplet state using the same methodology and functional as Oher et al., using their optimized structures as initial guesses. CASSCF calculations were then performed for the ground-state (S0) and excited-state (T1) geometries. An active space of 2 electrons in 9 orbitals, herein CAS(2,9), was chosen initially, which included the 7 5f orbitals as well as the σ bonding and antibonding “yl” orbitals, where 45 singlet and 36 triplet roots were considered, followed by mixing with SOC. These initial results showed that the lowest-lying spin–orbit excitation is S0 yl-σ to T1 5f_δ_ (Figure 2) at 16 800 cm^–1^. However, after these calculations we found that the active space had significantly changed from our original selection. For the singlets, many unoccupied 5f orbitals had rotated out (including 5f_δ_), in favor of unoccupied ligand orbitals, while some 5f orbitals remained in the triplet states (specifically, the occupied 5f_δ_). Because the active spaces differed considerably between spin multiplicities, the amount and nature of electron correlation is different, which is not ideal for the evaluation of transition energies. Hence, in an attempt to maintain a consistent active space, we attempted to expand the active space using RASSCF methods. We added the closest-energy secondary orbitals (uranium 6d orbitals) to RAS3, allowing a maximum of 2 electrons in this space, while the oxygen 2p orbitals were added to the RAS2 space for a total of 6 RAS2 orbitals with 12 electrons (RAS(12,0,2:0,6,11)). However, this was still not able to stabilize a consistent active space between singlet and triplet multiplicities, and larger active spaces quickly hit the computational limits of RASSCF.

To expand further, we attempted DMRG(16,17)SCF calculations and started with the largest feasible active space we obtained from RASSCF, consisting of the oxygen 2s and 2p orbitals as well as the uranium 5f (0, ±1, ±2) and 6d (±1, ±2) orbitals for both multiplicities at both geometries considering two roots per multiplicity to account for low-lying excitations. However, similar to the RASSCF calculations, the active space still differed between the singlet and triplet multiplicities. It was not until DMRG(16,40)SCF calculations that we could stabilize the 5f_δ_ orbitals in the active space in both spin multiplicities: these calculations were incredibly time-consuming and so were not pursued further. Comparing the vertical S0–T1 absorption energy calculated with DMRG(16,17)SCF to that found in the literature^39^ (Table 1), we find our result to be ca. 5000 cm^–1^ above the experimental and TD-DFT values. However, the nature of our transition is very similar.

**Table 1:** Vertical Absorption Energies for [UO_2_Cl_4_]^2–^ Calculated with DMRG(16,17)SCF and Compared to Experimental and TD-DFT Data^39^

	transition	experimental^39^	TD-DFT^39^	SOC-CASPT2^37^	DMRG(16,17)SCF
absorption/cm^–1^	σ_u_ (S0) → 5f_δ_ (T1)	20 096	20 737	20 280	25 207

Given these relatively poor results, we reasoned that our strategy of enlarging the active space was detrimental to the accuracy of the calculated excitation energy and, given the good accuracy of TD-DFT, that perhaps it could be more important to consider dynamical correlation. The latter is normally added on top of CASSCF calculations using CASPT2; however, this is not an option for DMRGSCF in OpenMolcas, so we opted to try MC-PDFT,^40^ which should be much faster than CASPT2. However, like other DFT methods, it is also dependent on the choice of the on-top functional, so some benchmarking is required. Therefore, MC-PDFT calculations were performed (Table 2) for each density functional available, four translated functionals (tLSDA, tPBE, tBLYP, and trevPBE) and their “fully-translated” variants^41^ (ftLSDA, ftPBE, ftBLYP, and ftrevPBE) on top of the DMRG(16,17)SCF calculations (Table 2). These calculations are reported spin-free as the addition of spin–orbit coupling does not change the energy of the vertical excitation.

**Table 2:** Vertical Absorption and Emission Energies for [UO_2_Cl_4_]^2–^ Calculated with DMRG(16,17)SCF-MC-PDFT, Compared to Experimental and TD-DFT Data^39^

	transition	experimental^39^	TD-DFT^39^	SOC-CASPT2^37^	DMRG(16,17)SCF	tPBE	tLSDA
absorption/cm^–1^	σ_u_ (S0) → 5f_δ_ (T1)	20 096	20 737	20 280	25 207	21 964	22 492
emission/cm^–1^	5f_δ_ (T1) → σ_u_ (S0)	-	19 924	-	23 686	21 248	21 854

		tBLYP	trevPBE	ftBLYP	ftrevPBE	ftPBE	ftLSDA
absorption/cm^–1^	σ_u_ (S0) → 5f_δ_ (T1)	21 848	21 874	23 183	23 046	23 187	23 839
emission/cm^–1^	5f_δ_ (T1) → σ_u_ (S0)	21 075	21 158	22 202	22 126	22 268	23 073

The addition of MC-PDFT greatly improves the agreement with the experimental data, with the translated functionals performing slightly better than their fully translated variants. This suggests that dynamical correlation is more important when calculating transition energies than obtaining a commensurate active space between spin multiplicities. While the use of MC-PDFT requires the choice of the functional such as for other DFT methods, we found that all translated functionals give very similar results so that the choice is not extremely important. It is clear, however, that our results are no more accurate than TD-DFT, and hence the benefits of multiconfigurational methods are not necessarily required or realized in U(VI) compounds.

### 3.2 [U(TREN^TIPS^)(N)]^−^ – U(V)

**Figure 3:**
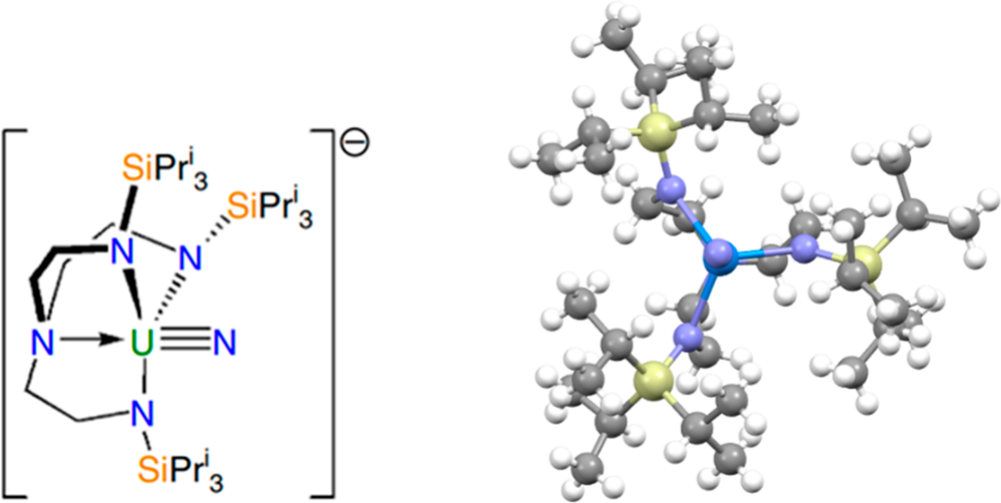
2D (left, reproduced from ref (42) under a CC BY 4.0 license) and 3D (right) molecular structure of [U(Tren^TIPS^)(N)]^−^. Purple = nitrogen, yellow = silicon, blue = uranium, and gray = carbon.

Uranium(V) has a single 5f electron in the ground state, which may require multiconfigurational methods in cases of high symmetry where some 5f orbitals are degenerate, and SOC must be considered to be a non-negligible perturbation to the electronic structure. Here we have chosen to look at [U(TREN^TIPS^)(N)]^−^ (Figure 3), studied previously by King et al.,^42^ for which experimental and CASSCF excitation energies are available. In this case, we are interested in the low-energy f–f absorptions between the degenerate spin–orbit doublet ground state to the excited spin–orbit doublet states. The ground doublet is either *m*_*J*_ = ±3/2 or ±5/2, as derived from the doubly degenerate orbital pairs of *m*_*l*_ = ±2 or ±3, respectively, which are rather close in energy, necessitating a multiconfigurational approach with SOC. Therefore, TD-DFT is not an appropriate method for obtaining excitation energies here. The choice of the starting active space is simple, considering the seven 5f orbitals (CAS(1,7)SCF) in the original work.

**Figure 4:**
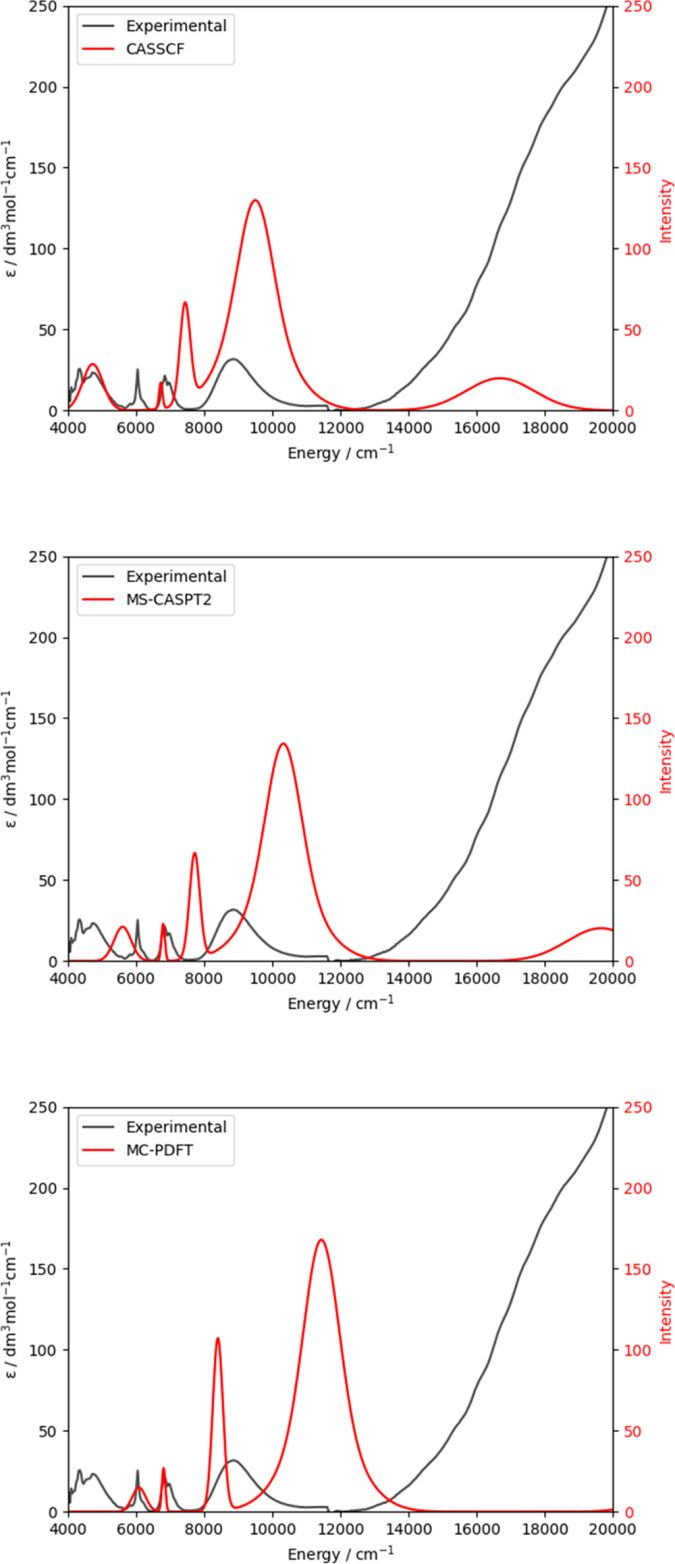
Absorption spectra for [U(Tren^TIPS^)(N)]^−^ calculated with CAS(1,7)SCF (top), CAS(1,7)SCF-MS-CASPT2 (middle), and CAS(1,7)SCF-MC-PDFT (bottom) compared to the experiment.^42^ Theoretical line widths are scaled to match the experimental peak widths.

As the experimental data are solution-phase, we have optimized the structure using the crystal structure as a starting point and then performed a CAS(1,7)SCF calculation for seven doublets (Table 3); we note that DFT optimization is often an acceptable approach for structural optimization, which is not very sensitive to orbital degeneracy and SOC effects.^43^ Unsurprisingly, the results are very similar to the original CAS(1,7)SCF values, where the differences (ca. 100 cm^–1^) are due to changes in the optimized geometry (cf. the crystal structure). As we found MC-PDFT corrections to be valuable for the U(VI) example above, we added them on top of the CAS(1,7)SCF results using the tPBE functional. However, in this case the agreement with the experimental data worsened, considerably overestimating the energies of the doublets (Table 3). As the active space is very small, CASPT2 calculations are affordable, and thus we used single-state (SS), multistate (MS), and extended multistate (XMS) variants to provide further estimates of the dynamical correlation (Table 3). In every case, dynamical correlation worsened the agreement with the experimental data over the CASSCF results (Figure 4).

**Table 3:** Spin-Orbit Doublet Energies of [U(Tren^TIPS^)(N)]^−^ from CAS(1,7)SCF-CASPT2 Calculations for Singlet-State, Multistate, and Extended Multistate Methods Compared to the Experimental Data^42^

CAS(1,7)SCF	MC-PDFT (tPBE)	SS-CASPT2	MS-CASPT2	XMS-CASPT2	experimental
759	858	742	966	1025	
4724	6083	5355	5616	5923	4700
6725	6807	6692	6751	6779	6000
7439	8401	8155	7788	7970	6900
9502	11 443	10 813	10 342	10 792	8900
16 690	22 488	20 601	19 706	20 568	18 000

In this case, the addition of dynamical correlation did not improve the agreement with experimental data. To try to improve further, we expanded the active space by adding the nitride 2p orbitals (RAS(7,1,1;3,7,3), seven roots) restricted to only single excitations. The agreement with the experimental data worsened (Table 4), which could indicate a poor choice of active space. Further inclusion of the 2p_*x*/*y*_ orbitals from the equatorial nitrogen donor atoms and the 2p_*z*_ orbital from the axial nitrogen donor in a RAS(21,1,1;10,7,10) calculation leads to even worse agreement, except for the highest energy state. This is likely because the highest energy state is the *m*_*J*_ = ±1/2 doublet arising from the *m*_*l*_ = 0 function which is formally the σ antibonding U-nitride orbital. Further expansions exceed computational limitations for RASSCF, and using seven roots is beyond the scope of the DMRG implementation in OpenMolcas, which is advisible only for the lowest few roots.

**Table 4:** Results Obtained in cm^–1^ from the RAS(7,1,1;3,7,3) and RAS(21,1,1;10,7,10) Calculations on [U(Tren^TIPS^)(N)]^−^ Compared to the CAS(1,7) Calculation and Experimental Data^42^

experimental	CAS (1,7)	RAS (7,1,1;3,7,3)	RAS (21,1,1;10,7,10)
	0	0	0
	759	800	863
4700	4724	4987	5082
6000	6725	6659	6966
6900	7439	7451	7704
8900	9502	9668	9916
18 000	16 690	17 096	17 319

Thus, we can conclude that the discrepancies between experimental and calculated excitation energies arise here from either extensive correlation effects that cannot be captured by the perturbative methods attempted or, more likely, from solvent effects such as screening, polarization, and geometric changes. Two methodologies to overcome these solvent effects would be to either perform molecular dynamics calculations or include explicit solvent molecules in a structural optimization.

### 3.3 [UCl_5_(THF)]^−^ – U(IV)

**Figure 5:**
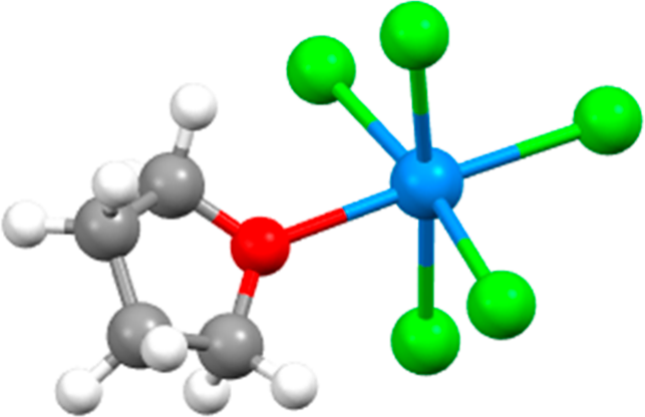
Molecular structure of [UCl_5_(THF)]^−^. Red = oxygen, green = chlorine, and blue = uranium.

Further increasing in complexity, uranium(IV) has a 5f^2^ ground configuration, meaning that there are two electrons in the 5f orbitals, leading to a ground-state triplet (^3^H_4_). The extra electron considerably complicates the electronic spectrum but also moves the electronic structure into a regime more akin to the trivalent lanthanide ions. Again, we consider only the f–f transitions; however, this time the excited states have both singlet and triplet multiplicities. Previous work by Hashem et al.^44^ studied the emission and absorption of uranium(IV) compounds experimentally and computationally using CASSCF and CASPT2. In their work, they investigated the f–d and LMCT transitions and concluded that the emission spectra of simple U(IV) compounds could be used as a diagnostic tool to deconvolute experimental luminescence spectra in the presence of [UO_2_]^2+^. For one complex in particular, [UCl_5_(THF)]^−^ (Figure 5), they provide a fully assigned experimental absorption spectrum for the f–f transitions, thus providing a useful benchmark.

**Figure 6:**
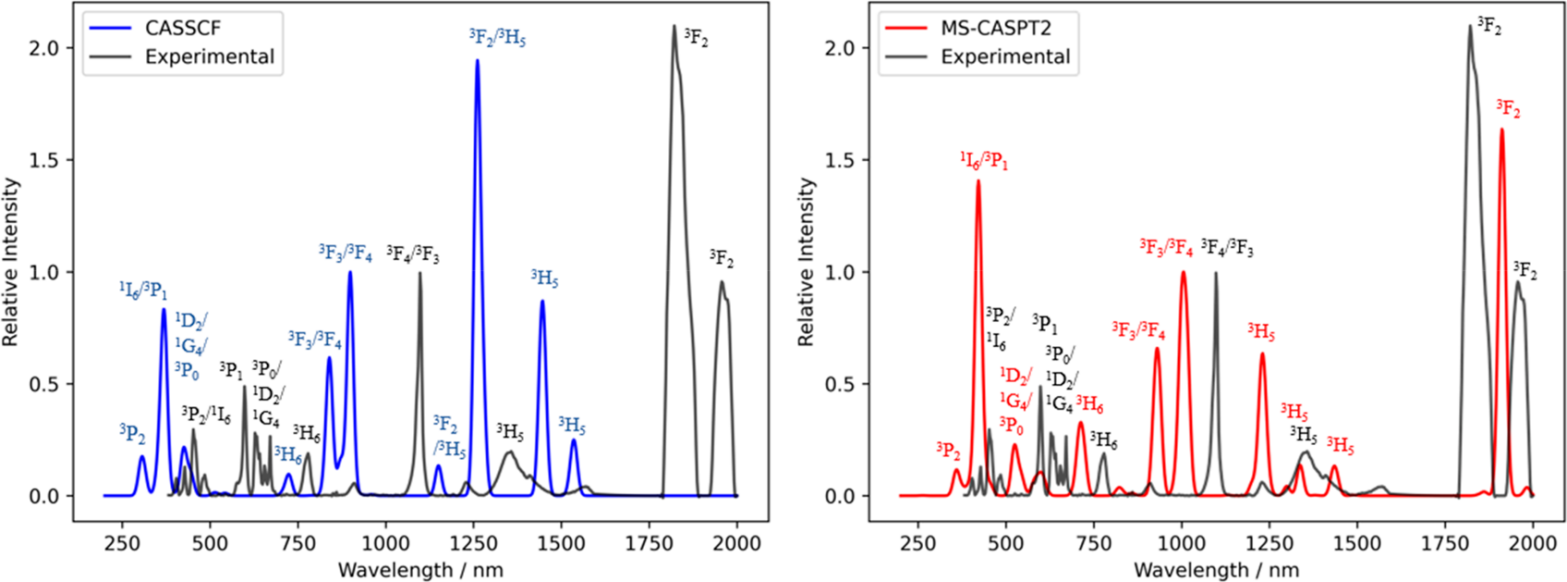
Absorption spectra of [UCl_5_(THF)]^−^ calculated by (left) CAS(2,7)SCF and (right) CAS(2,7)SCF-MS-CASPT2, compared to the experimental data (black). All spectra are normalized to the intensity of the ^3^F_3_/^3^F_4_ transition, and the calculated spectra are plotted with a FWHM line width of 9 nm.

Starting with a minimal active space CAS(2,7)SCF and considering 28 singlet and 21 triplet roots, we have followed up with MS-CASPT2 and MC-PDFT(tPBE) calculations. The spectra were calculated on the basis of CASSCF/CASPT2 isotropic Einstein coefficients after spin–orbit coupling and were assigned purely *ab initio* by transforming the spin–orbit states into a basis of well-defined spin, orbital, and total angular momentum by successive block diagonalization of the appropriate operators^45^ (Figure 6). Both the CAS(2,7)SCF and CAS(2,7)SCF-MS-CASPT2 calculations generally show good agreement with the experimental data (Figure 6), but the CAS(2,7)SCF-MC-PDFT results differ significantly (Figure S1). This is likely because MC-PDFT is a single-state method, which does not model how the dynamic correlation perturbation induces the interaction between nearly degenerate states in the f^2^ configuration.^46^ XMS-CASPT2 and extended dynamically weighted CASPT2^47^ (XDW-CASPT2) calculations were also performed (Figures S2 and S3), although they did not agree with the experimental data as well as MS-CASPT2 calculations.

The ^3^H_5_ peak in the experimental spectrum is very broad (1300–1500 nm) due to crystal field splitting, and the appearance of multiple peaks in both CASSCF (∼1200–1600 nm) and MS-CASPT2 (∼1200–1450 nm) calculations is simply due to an arbitrary line width being chosen to produce the theoretical spectrum, though the calculated crystal field splitting is undoubtedly imperfect. In the CASSCF spectrum, some of these peaks have a moderate contribution from the ^3^F_2_ state which is incorrect in comparison to the experimental data, which shows the ^3^F_2_ states between 1800 and 2000 nm; MS-CASPT2 calculations appear to correct this. The main ^3^F_3_/^3^F_4_ peak in the experiment occurs at 1100 nm, whereas in CASSCF this peak appears at ∼900 and 1000 nm for MS-CASPT2. While the theoretical spectra have two peaks corresponding to these states, the experimental spectrum only has one, although there is a small unlabeled peak at ca. 900 nm which could have some ^3^F_3_/^3^F_4_ contribution. The states at lower wavelengths are generally blue-shifted with respect to experiment: the ^1^I_6_ state is shifted 100 nm lower for CASSCF and 30 nm lower for MS-CASPT2 than its experimental position. These data clearly show that corrections for dynamical correlation are important when calculating the electronic transitions in U(IV) complexes.

While the peak positions are generally in good agreement for the MS-CASPT2 calculations, the relative intensities are not as accurate. For some features such as the ^1^D_2_ and ^3^H_6_ transitions, the relative intensities are in good agreement, but for others such as ^1^I_6_/^3^P_1_ and one of the ^3^H_5_ peaks, the intensities are much greater than the experimental data.

## 4 Conclusions

Herein we have calculated the absorption spectra for uranium compounds in the +4, +5, and +6 oxidation states using different levels of theory. We have found that DMRGSCF is a good alternative to CASSCF for larger active spaces, but when used alone it is not able to accurately calculate transition energies for U(VI), implying that dynamical correlation is crucially important. It seems that DMRGSCF-MC-PDFT approaches are just as accurate as TD-DFT in this case; however, the single-state MC-PDFT method is not appropriate in cases of orbital degeneracy, such as the U(VI) example herein, where CASPT2 approaches are more robust. This conclusion could be altered by adopting state-interaction pair-density functional theory methods^46^ in future work. In the case of U(V) [U(Tren^TIPS^)(N)]^−^, neither enlarging the active space nor adding dynamical correlation (by any means) was able to improve agreement with the experimental data over minimal CAS(1,7)SCF, suggesting that solvent effects must be crucial in this case. It is likely that this is generally true of U(V) compounds, where crystal field effects are on the same level of importance as spin–orbit coupling and electron correlation. While accurate environmental effects appear to be less crucial for the structure of U(IV) absorption spectra, we caution that the details of the fine structure contain compound-specific information, and hence we suggest that a crucial aspect of the calculation of uranium electronic spectra for U(V) and U(IV) must be the inclusion of an environmental model. We acknowledge that this is, computationally, a very costly predicament. Overall, it is clear that calculating electronic transitions accurately for actinide complexes using multiconfigurational methods is not a trivial task, and work must continue to find a generally reliable and consistent approach across different oxidation states and compounds. Furthermore, this work highlights the difficulty in accurately reproducing electronic spectroscopy in solution using the current state-of-the-art methods.

## Supporting Information Available

Absorption spectra of [UCl_5_(THF)]^−^ (calculated and compared to experimental data) (PDF)
