# Supplementary material for: Investigation of the Electronic Structure and Optical Spectra of Uranium (IV), (V), and (VI) Complexes Using Multiconfigurational Methods
Source: J Phys Chem A. 2022 Sep 6;126(36):6059–66. doi: 10.1021/acs.jpca.2c03314 (PMC9483975; doi:10.1021/acs.jpca.2c03314)
Supplement: Supplementary file 1 — jp2c03314_si_001.pdf [file jp2c03314_si_001.pdf]

Supplementary Information:

Investigation of the Electronic Structure and Optical Spectra of Uranium (IV), -(V) and -(VI) Complexes Using Multi-Configurational Methods

Michael Godsall[1] and Nicholas F. Chilton[1,*]

[1] Department of Chemistry, The University of Manchester, Manchester, M13 9PL, UK

List of Figures

Figure S1: Absorption spectra of [UCl5(THF)]- calculated by CAS(2,7)SCF-MC-PDFT, compared to the experimental data (black). All spectra are normalised to the intensity of the 3F3/3F4 transition, and the calculated spectra is plotted with a FWHM linewidth of 9 nm. S-3
Figure S2: Absorption spectra of $[UCl_5(THF)]^-$ calculated by CAS(2,7)SCF-XMS-CASPT2, compared to the experimental data (black). All spectra are normalised to the intensity of the $^3F_3/^3F_4$ transition, and the calculated spectra is plotted with a FWHM linewidth of 9 nm...S-4
Figure S3: Absorption spectra of $[UCl_5(THF)]^-$ calculated by CAS(2,7)SCF-XDW-CASPT2, compared to the experimental data (black). All spectra are normalised to the intensity of the $^3F_3/^3F_4$ transition, and the calculated spectra is plotted with a FWHM linewidth of 9 nm...S-5

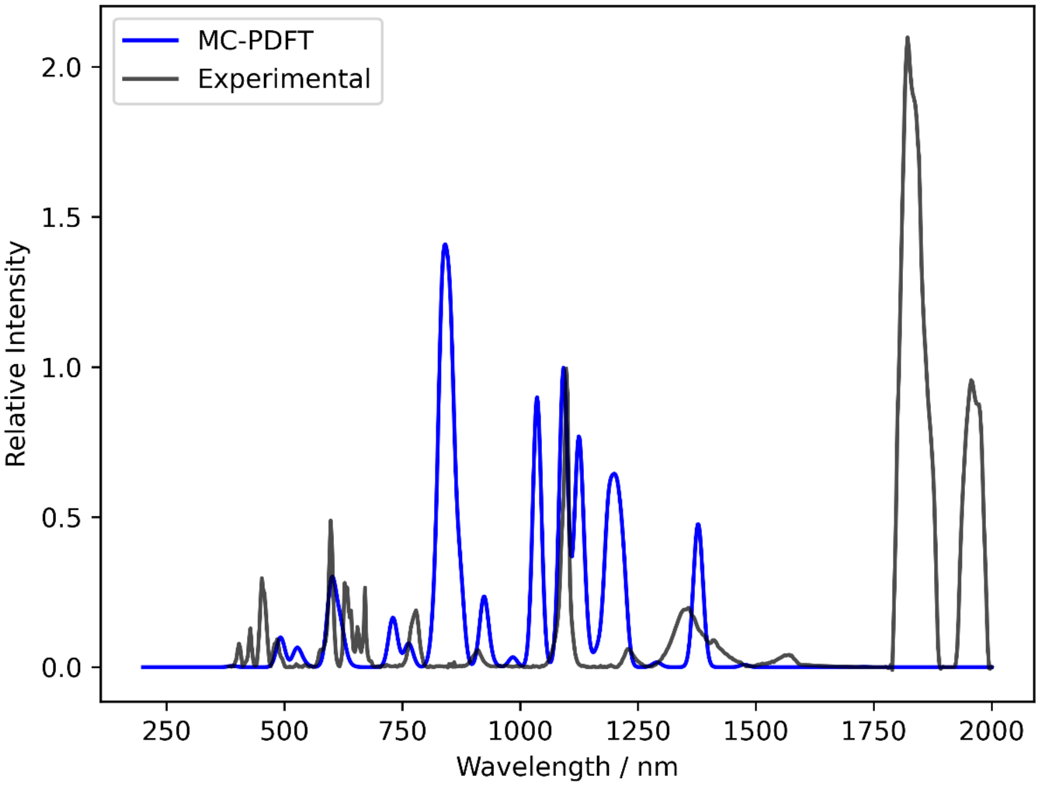

Figure S1: Absorption spectra of [UCl5(THF)]- calculated by CAS(2,7)SCF-MC-PDFT, compared to the experimental data (black). All spectra are normalised to the intensity of the 3F3/3F4 transition, and the calculated spectra is plotted with a FWHM linewidth of 9 nm.

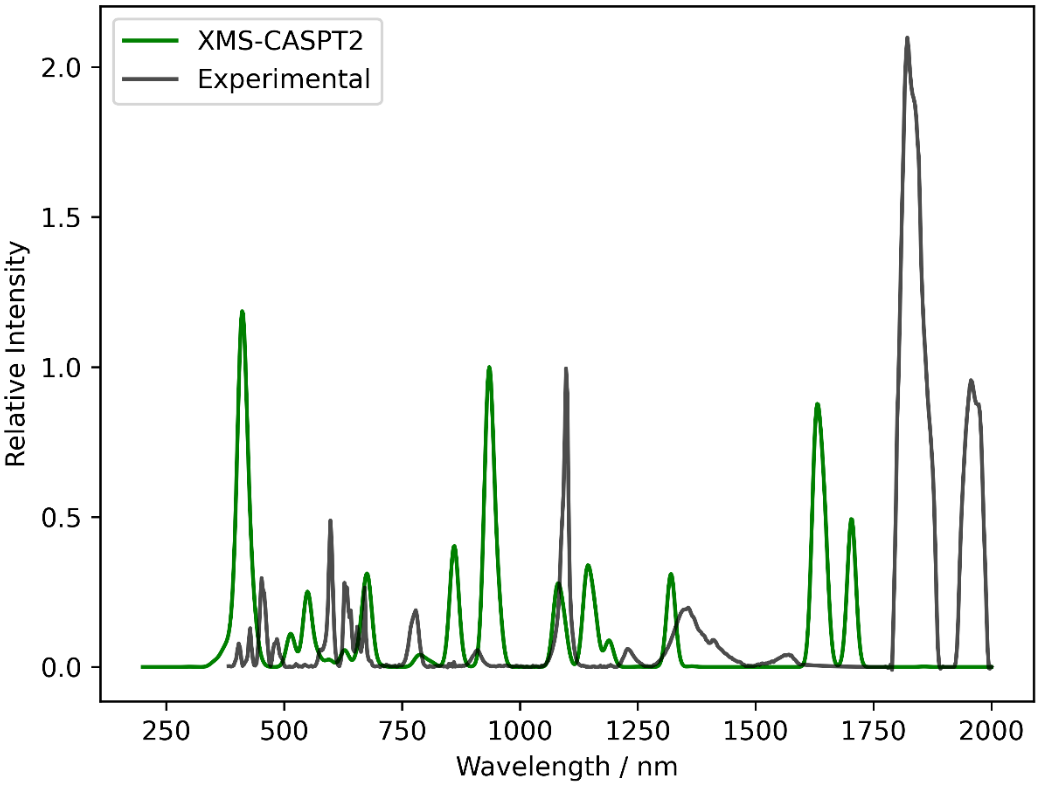

Figure S2: Absorption spectra of $[UCl_5(THF)]^-$ calculated by CAS(2,7)SCF-XMS-CASPT2, compared to the experimental data (black). All spectra are normalised to the intensity of the $^3F_3/^3F_4$ transition, and the calculated spectra is plotted with a FWHM linewidth of 9 nm.

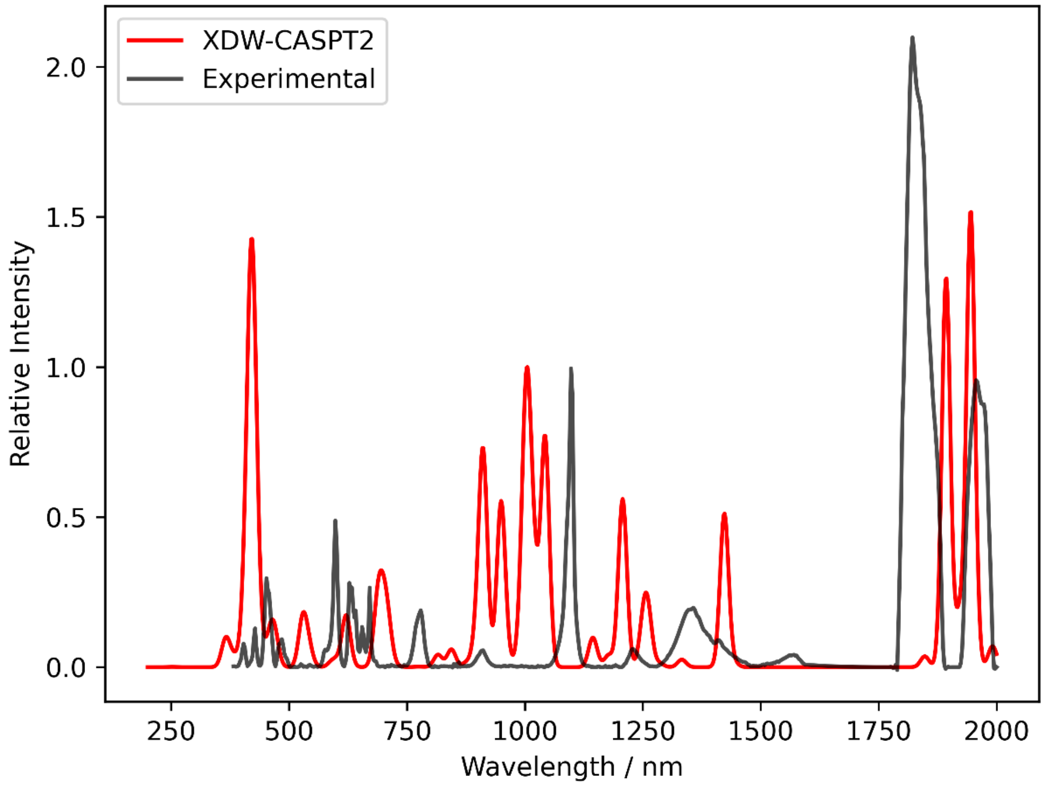

Figure S3: Absorption spectra of $[UCl_5(THF)]^-$ calculated by CAS(2,7)SCF-XDW-CASPT2, compared to the experimental data (black). All spectra are normalised to the intensity of the $^3F_3/^3F_4$ transition, and the calculated spectra is plotted with a FWHM linewidth of 9 nm.
